# A method for three-dimensional quantification of vascular smooth muscle orientation: application in viable murine carotid arteries

**DOI:** 10.1007/s10237-015-0699-4

**Published:** 2015-07-15

**Authors:** Bart Spronck, Remco T. A. Megens, Koen D. Reesink, Tammo Delhaas

**Affiliations:** Department of Biomedical Engineering, CARIM School for Cardiovascular Diseases, Maastricht University, Universiteitssingel 50, Room 3.359, 6229 ER Maastricht, The Netherlands; Institute for Cardiovascular Prevention, Ludwig-Maximilians-Universität, Pettenkoferstraße 9, 80336 Munich, Germany

**Keywords:** 3D smooth muscle cell orientation distribution, Arterial helix, Bingham distribution, Map projection, Two-photon laser scanning microscopy

## Abstract

When studying in vivo arterial mechanical behaviour using constitutive models, smooth muscle cells (SMCs) should be considered, while they play an important role in regulating arterial vessel tone. Current constitutive models assume a strictly circumferential SMC orientation, without any dispersion. We hypothesised that SMC orientation would show considerable dispersion in three dimensions and that helical dispersion would be greater than transversal dispersion. To test these hypotheses, we developed a method to quantify the 3D orientation of arterial SMCs. Fluorescently labelled SMC nuclei of left and right carotid arteries of ten mice were imaged using two-photon laser scanning microscopy. Arteries were imaged at a range of luminal pressures. 3D image processing was used to identify individual nuclei and their orientations. SMCs showed to be arranged in two distinct layers. Orientations were quantified by fitting a Bingham distribution to the observed orientations. As hypothesised, orientation dispersion was much larger helically than transversally. With increasing luminal pressure, transversal dispersion decreased significantly, whereas helical dispersion remained unaltered. Additionally, SMC orientations showed a statistically significant ($p < 0.05$) mean right-handed helix angle in both left and right arteries and in both layers, which is a relevant finding from a developmental biology perspective. In conclusion, vascular SMC orientation (1) can be quantified in 3D; (2) shows considerable dispersion, predominantly in the helical direction; and (3) has a distinct right-handed helical component in both left and right carotid arteries. The obtained quantitative distribution data are instrumental for constitutive modelling of the artery wall and illustrate the merit of our method.

## Introduction

Smooth muscle cells (SMCs) play a crucial role in regulating arterial vessel tone. When an SMC contracts, it exerts a force along its long axis. Therefore, the orientation of SMCs within the artery wall is mechanically of importance. Several mechanical models of the artery wall include a smooth muscle component, e.g. Masson et al. (2011), Spronck et al. (2015), Zulliger et al. (2004). To our knowledge, all currently available constitutive models assume SMC orientation to be strictly circumferential and ignore any dispersion in SMC orientation. In order to develop constitutive models that describe SMC orientation more realistically, knowledge on SMC orientation and its dispersion is essential.

The assumption in current constitutive models of strictly circumferentially oriented SMCs may have arisen from experimental studies (Peters et al. 1983; Walmsley 1983), stating that SMC orientation is circumferential. Other studies have shown a more disperse SMC orientation with two main orientations (Holzapfel et al. 2002) and have shown that SMCs are enveloped by collagen bundles, leading to a parallel orientation of collagen and SMCs in the media of rat aortas (O’Connell et al. 2008). In the aforementioned studies on SMC orientation, arteries were first fixed and subsequently histologically sectioned, potentially causing artefacts. To overcome this problem, a method using intact arteries is required.

Previously, we developed a two-dimensional (2D) method to quantify SMC orientation in vessels using two-photon laser scanning microscopy (TPLSM) (Spronck et al. 2014b). This method, which was used in rat ureters, had the advantage over previous methods that it did not require sectioning. However, it only yielded 2D orientations, neglecting out-of-plane orientation. Clearly, using such a method, it is impossible to quantify transverse angles. In addition, due to the curvature of the wall, only a very limited part of the wall could be analysed, leading to the inclusion of only a few SMCs per acquisition. To parameterise a constitutive model, ideally, three-dimensional (3D) orientation and dispersion information are available.

We hypothesised that SMC orientation would show considerable dispersion, based on recent published work assessing porcine aortic SMC orientation in 2D (Tonar et al. 2015). As we expected SMCs to be mainly oriented in the plane of the arterial wall (Peters et al. 1983), we further hypothesised SMC orientation dispersion in the helical direction to be greater than in the transversal direction. To test these hypotheses, we developed a method for the quantification of 3D SMC orientation, applicable to viable, intact murine carotid arteries. This 3D method does allow for the quantification of transverse angles and is suited for the analysis of a much larger part of the acquired vessel wall than the previous 2D method (Spronck et al. 2014a, b). In the present study, we used this 3D method to assess orientation and dispersion differences between left and right carotid arteries (potentially relevant from a developmental biology point of view). Additionally, we investigated the effect of increasing luminal pressure on 3D SMC orientation.

## Methods

### Animals and staining

**Fig. 1:**
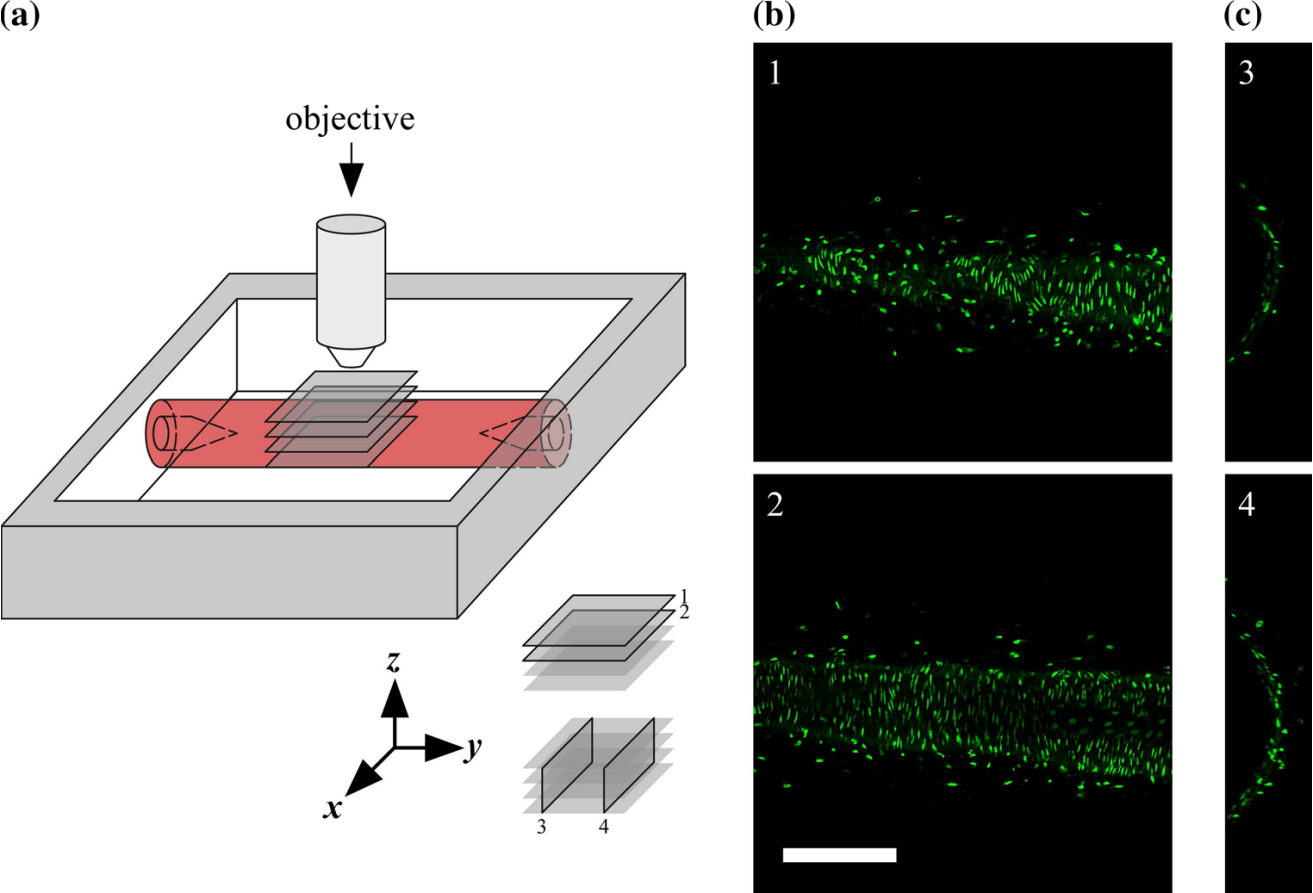
Data acquisition. **a** Measurement set-up. The common carotid artery is mounted between glass micropipettes and imaged with an upright microscope. *1* and *2* indicate example *x*–*y* image slices in **b**, and *3* and *4* indicate examples of reconstructed *x*–*z* image slices in **c**. **b** Example *x*–*y* image slices acquired at increasing imaging depth, i.e. at decreasing *z*-coordinate (1: $z=93 \, {\upmu \hbox {m}}$, 2: $z=81 \, {\upmu \hbox {m}}$). *Scale bar* $200 {\upmu \hbox {m}}$. **c** Examples of reconstructed *x*–*z* image slices. Figure scale equal to scale in **b**

Mice were killed with an overdose of isoflurane (Forane, Baxter, Deerfield, IL, USA). Left and right common carotid arteries of ten male C57BL/6JRj mice were excised, labelled at the proximal end, and carefully mounted between micropipettes (Fig. 1a) to maintain viability for a prolonged period of time ($>$2 h) as described previously (Megens et al. 2007). Carotid artery sections of $~5 \, \mathrm{mm}$ were mounted, yielding a length of $~3 \, \mathrm{mm}$ of artery usable for imaging (i.e. without imaging one of the pipettes). Calcium-free HEPES buffer was used as submersion medium. SMC nuclei were stained using $2.7 \, \upmu \hbox {M}$ SYTO$^{\circledR }$ 13 (Life Technologies$^{\circledR }$, Gaithersburg, MD, USA) for 30 min. Arteries were maximally vasodilated by the addition of nitroprusside (Sigma-Aldrich, St. Louis, MO, USA) to a concentration of $10\,\upmu \hbox {M}$ and were imaged at room temperature. Animals were checked for situs inversus totalis (SIT) by the inspection of the location of the visceral organs; none of the animals showed SIT. All animal experiments were approved by the local authority.

### Image acquisition

Viable mounted arteries were imaged using an upright TPLSM system (Leica$^{\circledR }$ TCS SP5II MP, Leica Microsystems$^{\circledR }$, Mannheim, Germany) equipped with a Leica$^{\circledR }$ HCX APO L 20$\times$/1.00 W water dipping objective (Fig. 1a). Two-photon excitation was achieved using a pulsed Ti-Sapphire laser (Spectra Physics$^{\circledR }$ Mai Tai$^{\circledR }$ DeepSee, Newport, USA) tuned at $825 \, \mathrm{nm}$. Signal detection was performed using a descanned hybrid diode detector (GaAsP) between wavelengths of 510 and $555 \, \mathrm{nm}$. Image slices of $1480\times 1480$ pixels were acquired with a pixel size of $0.5\times 0.5 \, \upmu \hbox {m}^2$, resulting in a field of view of $0.74\times 0.74 \, {\mathrm{mm}^2}$. Pixel dwell time was $1.1 \, \upmu \hbox {s}$, and acquisition was performed at 12-bit precision. Acquisition of one slice took $~2.6 \, \mathrm{s}$. Slice spacing was $0.5 \, {\upmu \hbox {m}}$, resulting in an effective voxel size of $0.5\times 0.5\times 0.5 \, {\upmu \hbox {m}^3}$. All subsequent image processing (see following section) was performed at this resolution; no downsampling was performed. Image slices formed stacks of $334 \pm 30 \, \mathrm{slices}$ ($\mathrm{mean} \pm \mathrm{SD}$). Left carotid arteries were imaged at luminal pressures of 40, 80, and $100 \, \mathrm{mmHg}$, respectively. Right carotid arteries were imaged at $40 \, \mathrm{mmHg}$. At each luminal pressure, one image stack was acquired for each animal. Representative image slices are shown in Fig. 1b,c. All subsequent image processing was performed at full resolution, i.e. no downsampling was performed. All arteries were imaged directly after excision. A maximum of 2 h was required to complete the imaging of the left and right arteries of one mouse. Previously, we have shown that in this time span, mounted arteries remain viable (Megens et al. 2007).

### Image processing

#### Deconvolution and vesselness filtering

Raw image stacks were deconvolved using 10-pass adaptive blind 3D deconvolution (AutoQuant X2, MediaCybernetics$^{\circledR }$, Bethesda, MD, USA). Deconvolution of one stack took approximately $35 \, \mathrm{min}$ on a modern computer [Intel$^{\circledR }$ i7-2670QM quad-core central processing unit (CPU) at $2.20\,\mathrm{GHz}$ and $16 \, \mathrm{GB}$ of random access memory (RAM)]. Further data processing and analysis were performed using MATLAB$^{\circledR }$ R2014b (MathWorks$^{\circledR }$, Natick, MA). After deconvolution, the deconvolved data stack was 3D vesselness filtered (Frangi et al. 1998) to enhance elongated structures. Note that in contrast to the approach described in our previous paper (Spronck et al. 2014b), filtering is performed in 3D on the image stack as a whole. A $3\times 3$ Hessian matrix was calculated for each voxel using a Gaussian width of $\sigma _x = \sigma _y = \sigma _z = 1\,{\upmu \hbox {m}}$. Hessian components were calculated by convolving the image stack with discretised Gaussian derivative kernels. The eigenvalues of the Hessian matrix ($\lambda _1 \le \lambda _2 \le \lambda _3$) were used for performing the vesselness filtering. Vesselness parameters were $\alpha =\beta =0.5$ (Frangi et al. 1998). *c* was determined for each image stack as half the value of the maximum Hessian norm, as suggested by Frangi et al. (1998). Vesselness filtering of one stack took approximately $65 \, \mathrm{min}$ on a modern computer (see above). All further image processing steps were completed in $<$1 min per stack.

#### Clustering and cell orientation calculation

**Fig. 2:**
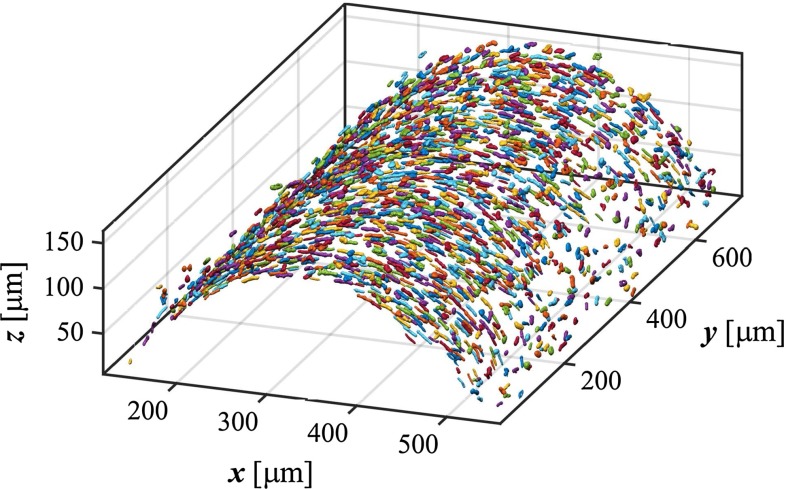
Isosurface plot showing detected SMC nuclei. Nuclei are randomly coloured for clarity. *SMC* smooth muscle cell

To separate cell nuclei from background, the vesselness stack was thresholded at a cut-off value of 0.01. Voxels were then clustered based on their 3D 6-connected neighbourhood (Gonzalez and Woods 2008). Clusters were included based on their volume using upper ($320.1 \, {\upmu \hbox {m}^3}$) and lower ($38.5 \, {\upmu \hbox {m}^3}$) thresholds calculated as $\mathrm{mean}\pm 2\mathrm{SD}$ from aortic SMC nucleus sizes (O’Connell et al. 2008). An example of a stack of clustered nuclei is given in Fig. 2. For each cluster, an inertia matrix was calculated (Jähne 1993; Vader et al. 2009). The eigenvector corresponding to the largest eigenvalue of this matrix represents the principal SMC orientation.

#### Coordinate transformation

**Fig. 3:**
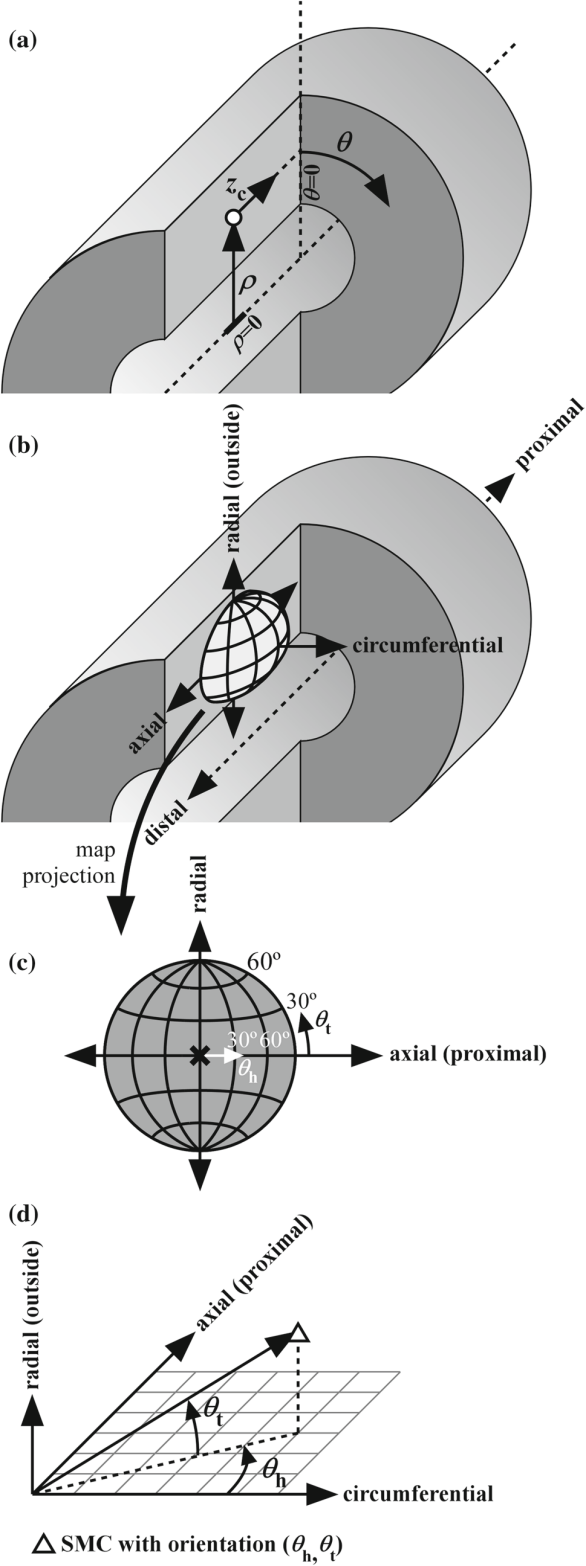
Coordinate and orientation definitions. **a** Definition of cylindrical coordinate system using radial ($\rho$), angular ($\theta$), and axial ($z_\mathrm{c}$) coordinates. **b, c** Orientations of SMCs can be visualised as points on a unit hemisphere. **b** The orientation of such a hemisphere with respect to the vessel. **c** The two spherical coordinates that are used to quantify SMC orientation. $\theta _\mathrm{h}$ and $\theta _\mathrm{t}$ are an SMC’s helix and transverse angles, respectively. **d** Exact definitions of $\theta _\mathrm{h}$ and $\theta _\mathrm{t}$. $\theta _\mathrm{h}$ is the angle that the orientation’s projection on the axial-circumferential plane makes with the circumferential direction. $\theta _\mathrm{t}$ is the angle that the orientation makes with the axial–circumferential plane. *SMC* smooth muscle cell

To assess SMC orientations with respect to the vessel wall, a cylinder was least-squares fitted through the detected centroids as follows: first, a rough fit was obtained by fitting a cylinder through all centroids. Using the axis of this cylinder, the inner layer of centroids was detected by selecting the centroids closest to the axis in a moving 3D search window of $50 \, {\upmu \hbox {m}}$ in axial and $10^{\circ }$ in angular directions. Second, a cylinder was fitted through the detected inner layer of centroids. The axis of this cylinder was used as a reference to convert all centroid locations to cylindrical coordinates ($\rho ,\theta ,z_\mathrm{c}$, Fig. 3a). Subscript c in $z_\mathrm{c}$ is used to distinguish the axial cylindrical coordinate ($z_\mathrm{c}$, Fig. 3a) from the vertical Cartesian coordinate (*z*, Figs. 1, 2). The orientations of the SMCs were expressed locally with respect to the vessel wall (Fig. 3b) and are represented as a point on a unit hemisphere (Fig. 3c). Orientations were quantified by a helix angle ($-90^{\circ } \le \theta _\mathrm{h} < 90^{\circ }$) and a transverse angle ($-90^{\circ } \le \theta _\mathrm{t} < 90^{\circ }$, Fig. 3c, d). Visual inspection of the detected cell nuclei (as exemplified in Fig. 2) shows that, from a certain imaging depth, the number of detected nuclei decreased. In order to assure that we imaged a region of sufficient image quality, centroids were selected from an angular region of interest of $-22.5^\circ \le \theta \le 22.5^\circ$. In order to prevent border effects, an axial window slightly smaller than the $z_{\mathrm{c}}$-range was manually selected ($704 \pm 23 \, {\upmu \hbox {m}}, \mathrm{mean} \pm \mathrm{SD}$). In six stacks, using this large axial window led to SMC layers getting merged during SMC layer detection (see following section) due to inhomogeneities in vessel diameter along the axial coordinate. In these cases, a smaller window of $379 \pm 74 \, {\upmu \hbox {m}}\,(\mathrm{mean} \pm \mathrm{SD}$) was used.

#### Smooth muscle layer separation

**Fig. 4:**
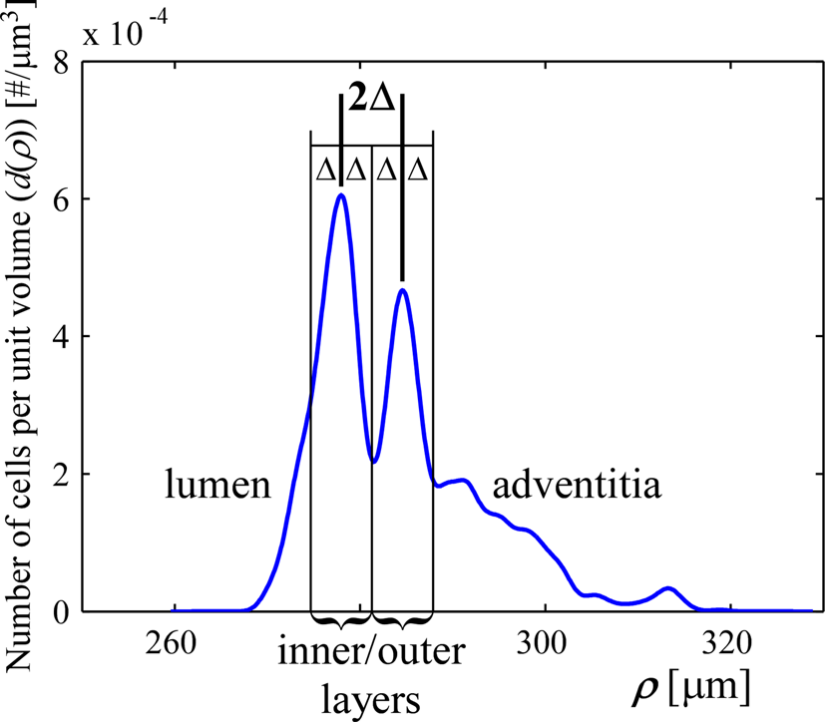
Representative example of SMC density as a function of the radial coordinate ($\rho$, Fig. 3a). Inner and outer layers of SMCs are clearly visible. Layers were detected by finding the two largest density maxima. The distance between those maxima was defined as $2\Delta$. SMCs are assumed to be part of the inner/outer layer if they are within a distance of $\Delta$ from their respective maximum. *SMC* smooth muscle cell

Cell density as a function of the radial coordinate ($\rho$) was calculated using kernel smoothing density estimation (KDE) (Silverman 1986). KDE allows for estimation and visualisation of the probability density from a set of data points. In our case, the KDE shows the density of SMCs at a certain radial coordinate ($\rho$). A Gaussian kernel with $\sigma =1 \, {\upmu \hbox {m}}$ was used on the list of radial coordinates ($\rho$) of all included nuclei of one stack, yielding the initial kernel density estimate $f(\rho ) \, [{{\upmu \hbox {m}}^{-1}}]$. This estimate was converted to an absolute density $d(\rho )$ in cells per ${\upmu \hbox {m}^3}$ via

1 $$\begin{aligned} d(\rho ) = f(\rho ) \frac{N}{r_{\theta } r_{z_\mathrm{c}} \rho } , \end{aligned}$$

with *N* the total number of detected nuclei, $r_{\theta }$ the angular region of interest in radians, and $r_{z_\mathrm{c}}$ the axial region of interest in ${\upmu \hbox {m}}$. An example density plot is shown in Fig. 4a. SMCs showed to be concentrated in two distinct layers, as visible in the example. We analysed SMC orientation separately for these (inner and outer) layers. To separate the layers, we detected the two density maxima, the distance between which we defined as $2\Delta$. Each layer is taken to be centred around its maximum, with boundaries of $\pm \Delta$ on each side (Fig. 4a). The artery diameter (as reported in “Results” section) is taken to be the middle between the inner and outer layer density maxima.

#### Orientations of SMC nuclei in detected layers

**Fig. 5:**
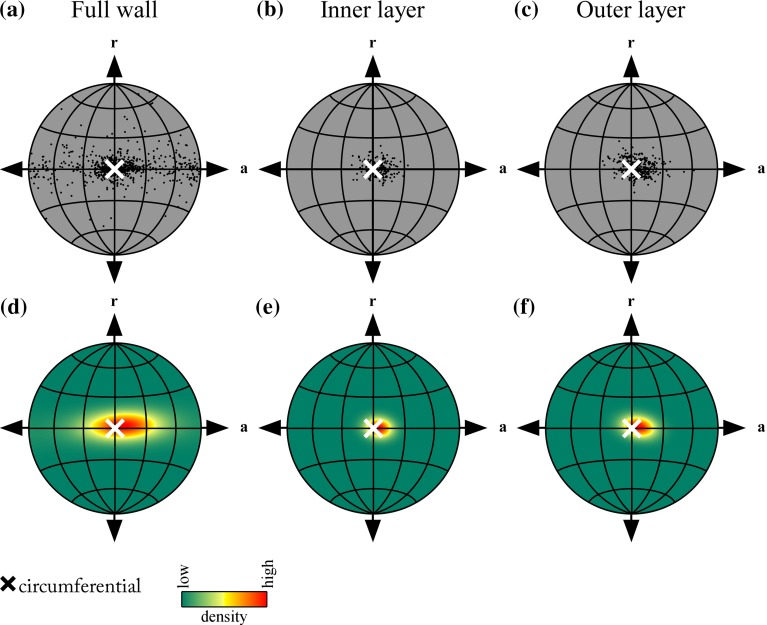
Representative examples of orientations of SMC nuclei in the artery wall. Orientations of detected nuclei, visualised using Lambert equal-area projection **a** before layer separation, **b** for the inner layer, and **c** for the outer layer. The Lambert equal-area projection is elaborated on in “Appendix 1”. **d–f** Corresponding fitted Bingham distributions to orientations in **a–c**. The obtained Bingham distributions provide a quantification of the distribution of the individual orientations. The distribution parameters are explained in Fig. 6. *SMC* smooth muscle cell

After smooth muscle layer separation, SMC orientations can be depicted on a projected hemisphere, as defined in Fig. 3c. A representative example of such a visualisation is given in Fig. 5a–c. The need for using a projection is elaborated on in “Appendix 1”.

### Orientation statistics

As our method yields orientations in 3D, appropriate, spherical statistics are required to accurately quantify our results (Fisher et al. 1993). In particular, the obtained orientations are undirected (i.e. axial data), requiring a 3D probability distribution that is antipodally symmetric (Mardia and Jupp 2000). One of the simplest distributions of this kind would be a (rotationally symmetric) Watson distribution (Mardia and Jupp 2000). However, this distribution requires data to be rotationally symmetric around the main direction. In the vessel wall, the largest spread in SMC direction will be in the axial–circumferential plane, whereas the spread in the radial–circumferential plane will be much smaller. The probability distribution used should be able to accommodate this non-rotationally symmetric geometry. The Bingham distribution fulfills this requirement, while still being antipodally symmetric (Bingham 1964, 1974).

The Bingham density function has the form (Bingham 1964):2where $\kappa _1$ and $\kappa _2$ are two concentration parameters,  and  are two orthogonal unit vectors indicating the two spread directions of the distribution (corresponding to $\kappa _1$ and $\kappa _2$), and  the current local (Cartesian) coordinate on the unit sphere. The normalisation function $d(\kappa _1,\kappa _2)$ was evaluated by numerically solving the integral (Bingham 1964; Onstott 1980)

3 $$\begin{aligned}&d(\kappa _1,\kappa _2) \nonumber \\&\quad = \! \int \limits _0^{2\pi } \! \int \limits _{0}^{\pi } \! \exp \! \left( \! \left( \kappa _1\cos ^2 \! \phi + \kappa _2\sin ^2 \! \phi \right) \sin ^2 \! \theta \right) \sin \theta \hbox { d}\theta \hbox { d}\phi .\nonumber \\ \end{aligned}$$

**Fig. 6:**
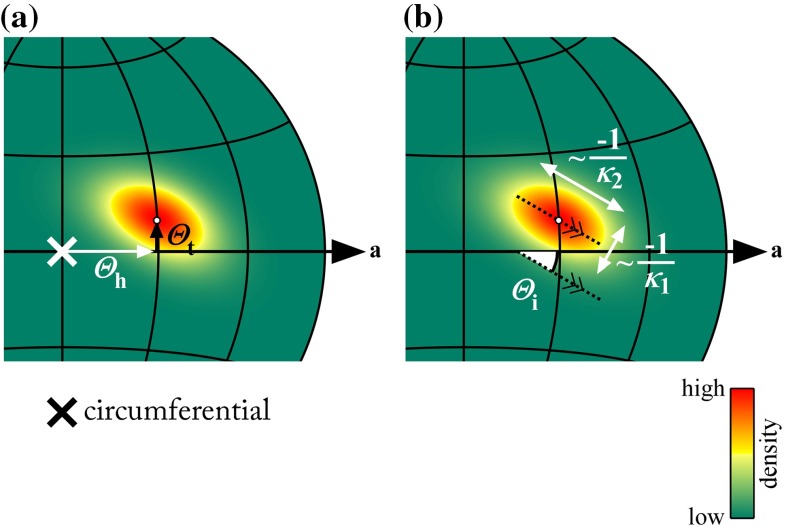
Definitions of Bingham distribution parameters. **a** Parameters describing the distribution’s centre. $\varTheta _\mathrm{h}$ and $\varTheta _\mathrm{t}$ are the centre’s helix and transverse angles. **b** Parameters describing the distribution’s dispersion. $\kappa _1$ and $\kappa _2$ describe the distribution’s concentration along two orthogonal directions. The more negative a $\kappa$ is, the narrower the distribution is in the respective direction (Onstott 1980). $\varTheta _\mathrm{i}$ is the angle that the principal spread direction (corresponding to $\kappa _2$) makes with the axial–circumferential plane. In this example, $\varTheta _\mathrm{h}=30^\circ$, $\varTheta _\mathrm{t}=10^\circ$, $\kappa _1=-30$, $\kappa _2=-10$, and $\varTheta _{\mathrm{i}}=-25^\circ$. *a* axial

Fitting the Bingham distribution to our data was performed in two steps by using the moment method (Bingham 1964; Onstott 1980; Tanaka 1999). First, the local centroid orientations were converted to Cartesian coordinates4and the following matrix was computed:5with *N* the number of detected centroids.  and  can now be obtained as the eigenvectors of  corresponding to the smallest and middle eigenvalues, respectively. , corresponding to the largest eigenvalue, represents the distribution’s centre. It is converted back to spherical coordinates ($\varTheta _\mathrm{h},\varTheta _\mathrm{t}$, Fig. 6a) using

6 $$\begin{aligned} \varTheta _\mathrm{h}= & {} \arctan \frac{\mu _{3,y}}{\mu _{3,x}} , \quad \hbox { and}\end{aligned}$$

7 $$\begin{aligned} \varTheta _\mathrm{t}= & {} \arctan \frac{\mu _{3,z}}{\sqrt{\mu _{3,x}^2 + \mu _{3,y}^2}}, \end{aligned}$$

where $\mu _{3,x}$, $\mu _{3,y}$, and $\mu _{3,z}$ represent the *x*, *y*, and *z* components of . Note the use of capital thetas, representing the orientation’s centre. $\varTheta _\mathrm{i}$ is defined as the angle that  makes with the *xy* plane (Fig. 6b). The second step in fitting the Bingham distribution is finding $\kappa _1$ and $\kappa _2$. This is performed through maximisation of the log-likelihood function (Bingham 1964; Onstott 1980; Tanaka 1999)

8 $$\begin{aligned} F = -N \ln 4\pi - N\ln d(\kappa _1,\kappa _2) + \kappa _1 \tau _1 + \kappa _2 \tau _2 , \end{aligned}$$

where $\tau _1$ and $\tau _2$ are the smallest and middle eigenvalues of . Maximisation was performed by the minimisation of $-F$ using the MATLAB Optimization Toolbox fmincon function and the interior-point algorithm, while constraining $\kappa _1$ and $\kappa _2$ to the interval $(-\infty ,0)$. Bingham distributions of the representative examples in Fig. 5a–c are shown in Fig. 5d–f.

## Results

### Qualitative results

Orientation plots for all arteries studied show a cloud of points that is approximately centred around the circumferential direction, as also visible in the representative example (Fig. 5a–c). Although these plots provide a good qualitative overview of the orientations of the SMC nuclei, it is hard to derive quantitative conclusions from them. Therefore, Bingham distributions were fitted to the acquired orientations, as exemplified in Fig. 5d–f, providing quantitative orientation data. These quantitative (Bingham parameter) results are discussed below.

### Left versus right carotid arteries

**Fig. 7:**
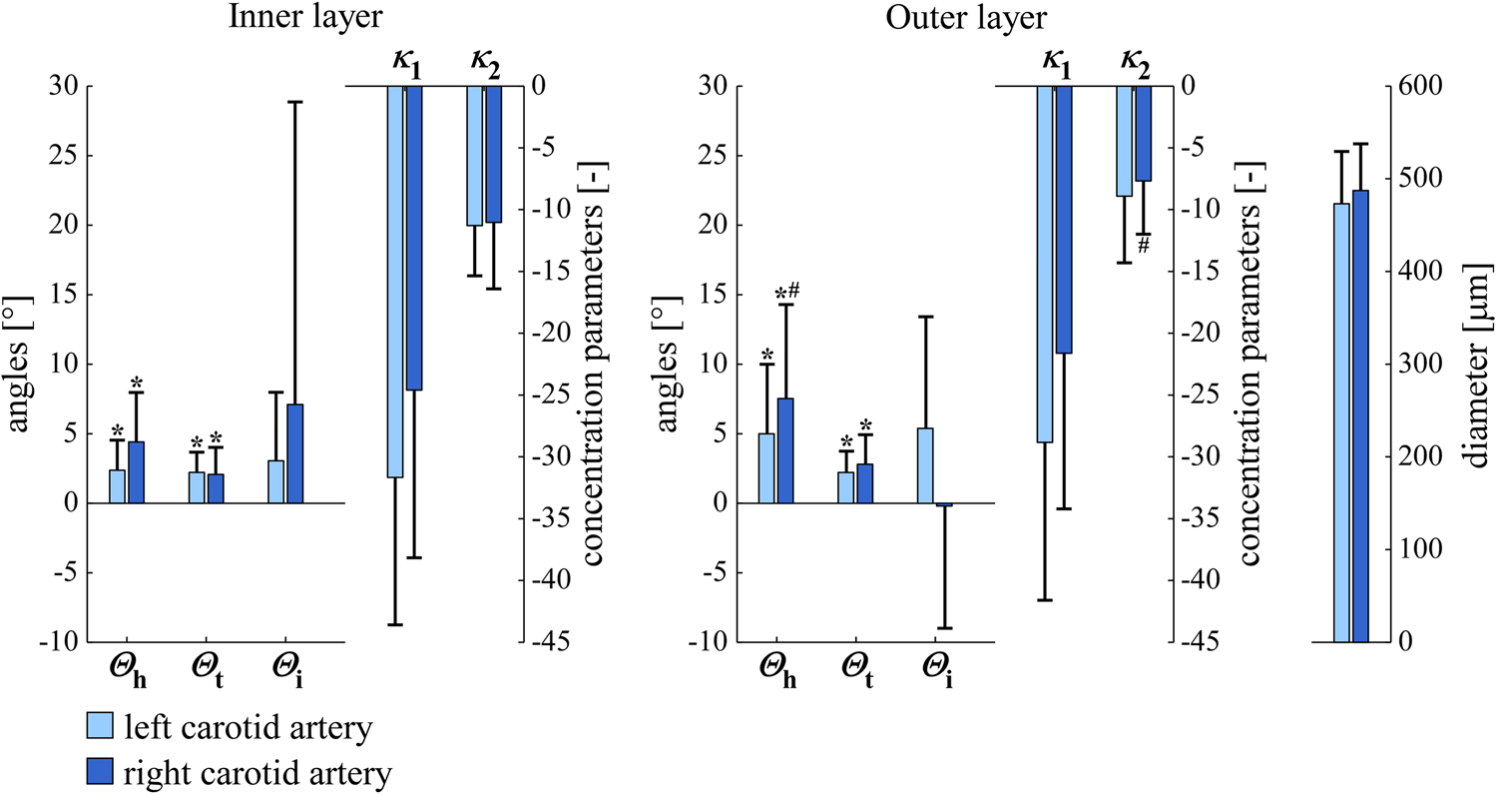
SMC orientations and diameters in *left* and *right* carotid arteries at $40 \, \mathrm{mmHg}$ ($\mathrm{circular \, mean} \pm \mathrm{circular \, SD}$ for $\varTheta _\mathrm{h}$, $\varTheta _\mathrm{t}$, and $\varTheta _\mathrm{i}$; $\mathrm{arithmetic \, mean} \pm \mathrm{SD}$ for $\kappa _1$, $\kappa _2$, and diameter). $^{*}p<0.05$ versus angle zero (Rayleigh test). $^\mathrm{\#}p<0.05$ inner versus outer layer (Rayleigh or paired Student’s *t* test). $\varTheta _\mathrm{h}$, $\varTheta _\mathrm{t}$, $\varTheta _\mathrm{i}$, $\kappa _1$, and $\kappa _2$ are defined in Fig. 6. *SD* standard deviation, *SMC* smooth muscle cell

Figure 7 shows Bingham parameters and diameters for left ($n=10$) and right ($n=10$) carotid arteries at a luminal pressure of $40 \, \mathrm{mmHg}$. Left and right artery diameters are nearly equal. As $\varTheta _\mathrm{i}$ is relatively close to zero, $\kappa _1$ represents dispersion in transversal direction and $\kappa _2$ represents dispersion in helical direction. The fact that $\kappa _1$ is more negative than $\kappa _2$ indicates that dispersion in helical direction is larger than in transversal direction. This holds for left as well as right arteries. $\varTheta _\mathrm{h}$ is positively different from zero in the right as well as the left arteries, in both the inner and outer layers (all $p<0.05$), which is indicative of a right-handed helical pattern. No statistically significant differences were found between left and right arteries, except that only in the right arteries, $\varTheta _\mathrm{h}$ was significantly larger in the outer layer than in the inner layer. $\varTheta _\mathrm{t}$ shows the same pattern, being significantly different from zero in all cases. Please refer to “Discussion” section for an interpretation of the latter finding.

### Dependence of smooth muscle orientation on luminal pressure

**Fig. 8:**
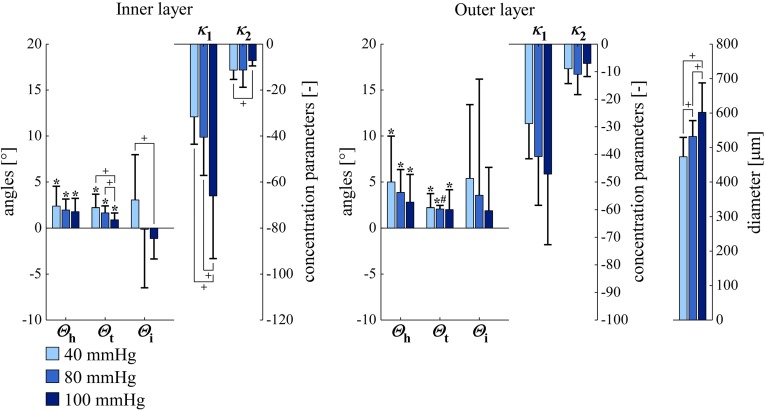
SMC orientations and arterial diameters in *left* carotid arteries at luminal pressures of 40, 80, and $100 \, \mathrm{mmHg}$ ($\mathrm{circular \, mean} \,\pm \, \mathrm{circular \, SD}$ for $\varTheta _\mathrm{h}$, $\varTheta _\mathrm{t}$, and $\varTheta _\mathrm{i}$; $\mathrm{arithmetic \, mean} \pm \mathrm{SD}$ for $\kappa _1$, $\kappa _2$, and diameter). $^{*}p<0.05$ versus angle zero (Rayleigh test). $^\mathrm{\#}p<0.05$ inner versus outer layer (Rayleigh or paired Student’s *t* test). $^\mathrm{+}p<0.05$ for parameter difference between pressures (Rayleigh or paired Student’s *t* test). $\varTheta _\mathrm{h}$, $\varTheta _\mathrm{t}$, $\varTheta _\mathrm{i}$, $\kappa _1$, and $\kappa _2$ are defined in Fig. 6. *SD* standard deviation, *SMC* smooth muscle cell

As expected, artery diameter increased significantly with increasing luminal pressure, illustrating vessel deformation at macro-level (Fig. 8). Figure 8 further shows Bingham parameters for the left carotid arteries ($n=10$) at three luminal pressures (40, 80, and $100 \, \mathrm{mmHg}$). $\varTheta _\mathrm{h}$ and $\varTheta _\mathrm{t}$ are both again significantly different from zero in all cases. $\kappa _1$ shows a very consistent pattern in the inner layer, becoming more negative with increasing pressure, thereby indicating a decrease in SMC orientation dispersion. As mentioned above, because $\varTheta _\mathrm{i}$ is relatively close to zero, this decrease is approximately along the transversal direction. $\kappa _2$ increases with increasing luminal pressure from 40 to $100 \, \mathrm{mmHg}$ in the inner layer, reflecting an increase in dispersion in helical direction. $\kappa _1$ and $\kappa _2$ show the same pattern with increasing luminal pressure in the outer layer, although it is not statistically significant here.

### Reproducibility

Reproducibility of our findings was assessed and is described in “Appendix 2”.

## Discussion

In this study, we quantified SMC orientation and dispersion in viable murine carotid arteries in full 3D. We found that SMCs are arranged in two layers, and we quantified SMC orientation and dispersion for each layer separately. Orientation dispersion in the helical direction was larger than in the transversal direction. We found a statistically significant right-handed helix in both left and right arteries, in both layers. With an increase in luminal pressure, the dispersion in transversal orientation was found to decrease.

The quantification method we developed in this study has three key advantages over previous methods: (1) arteries are mounted in their in vivo geometry and are kept viable (Megens et al. 2007; 2) by using TPLSM, 3D microscopic image stacks at large imaging depths (as compared to e.g. confocal laser scanning microscopy) can be acquired (Oheim et al. 2001); and (3) the present processing method analyses the full 3D image stack as a whole, instead of analysing data on a slice-by-slice basis as most other methods do [including our previous method (Spronck et al. 2014b)]. As arteries are mounted in their in vivo geometry (point 1), artefacts that could potentially arise due to fixation (e.g. shrinking) and/or sectioning are absent. It has been shown that the present vessel mounting and TPLSM imaging set-up (points 1,2) can be used to visualise both structural and functional properties of intact viable murine arteries. Its physiological relevance is demonstrated in various studies. Examples include the study of the endothelial glycocalyx (Reitsma et al. 2011; Slaaf and Zandvoort 2011), endothelial junctional adhesion molecule A (JAM-A) and its effect on monocyte or platelet recruitment (Karshovska et al. 2014; Schmitt et al. 2014), or the presence of smooth muscle progenitor cells (Subramanian et al. 2010) and proliferating endothelial cells (Schober et al. 2014) in healthy and atherosclerosis-prone arteries. Consequently, we believe that the results obtained in our study are relevant and well translatable to an in vivo situation, albeit that we studied mouse and not human arteries. In a slice-by-slice analysis, *flat* image slices are acquired of a *curved* object, causing cells at various depths of the wall to end up in one image slice (Spronck et al. 2014b). This limitation is overcome in our present approach by analysing the acquired image stacks as a whole and using a curved (cylindrical) coordinate system (point 3), omitting the need for a small region of interest (Spronck et al. 2014b). Hence, our 3D method allows a larger number of nuclei to be analysed and thus provides more robust results. Furthermore, transverse angles and dispersion therein can be analysed, whereas in a slice-by-slice analysis, out-of-plane information is lost.

**Fig. 9:**
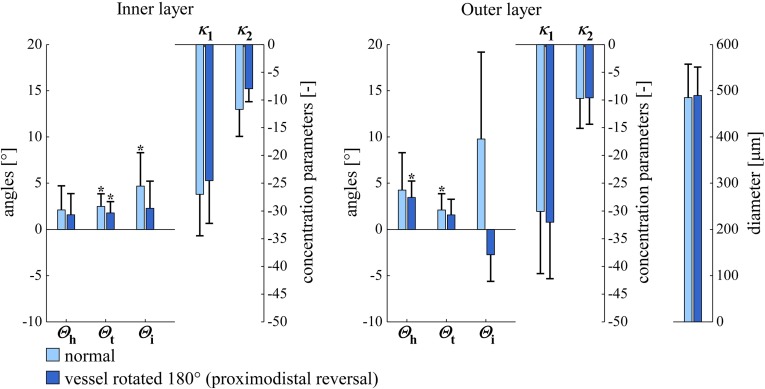
SMC orientations and arterial diameters in normal and rotated *left* carotid arteries at a luminal pressure of $40 \, \mathrm{mmHg}$ ($\mathrm{circular \, mean} \pm \mathrm{circular \, SD}$ for $\varTheta _\mathrm{h}$, $\varTheta _\mathrm{t}$, and $\varTheta _\mathrm{i}$; $\mathrm{arithmetic \, mean} \pm \mathrm{SD}$ for $\kappa _1$, $\kappa _2$, and diameter). $^{*}p<0.05$ versus angle zero (Rayleigh test). $\varTheta _\mathrm{h}$, $\varTheta _\mathrm{t}$, $\varTheta _\mathrm{i}$, $\kappa _1$, and $\kappa _2$ are defined in Fig. 6. *SD* standard deviation, *SMC* smooth muscle cell

Our results display statistically significant mean transverse and helix angles ($\varTheta _\mathrm{t}$ and $\varTheta _\mathrm{h}$, Figs. 7, 8). SMC orientation shows considerable dispersion in both helical and transversal directions. To critically evaluate our findings, we performed the following two additional experiments for five left arteries at a luminal pressure of $40 \, \mathrm{mmHg}$: (1) we *digitally* rotated our image stack (after deconvolution) by $180^\circ$ around the *z*-axis. (2) We *physically* rotated the mounting chamber by $180^\circ$ around the *z*-axis (i.e. around the objective axis). As compared to the non-rotated situation, both experiments should lead to a negation of $\varTheta _\mathrm{t}$ and $\varTheta _\mathrm{i}$. Experiment 1 indeed led to these negations (data not shown) and did not affect $\varTheta _\mathrm{h}$, $\kappa _1$, and $\kappa _2$. Experiment 2, on the contrary, still yielded positive $\varTheta _\mathrm{t}$ values (Fig. 9). The observed significant average transverse angle ($\varTheta _\mathrm{t}$) therefore seems to be a methodological artefact and not a true anatomical feature. We believe that this artefact results from inhomogeneities in image field brightness, slightly shifting the fitted cylinder axis and causing an artificially significant $\varTheta _\mathrm{t}$ value. The mean helix angle ($\varTheta _\mathrm{h}$) observed in these two experiments remained positive, as would be theoretically expected. For the physically rotated acquisitions, we used the same imaging region as used in these arteries for the non-rotated acquisitions. We achieved this by measuring the distance between the non-rotated imaging region and the proximal pipette (measuring the movement of the calibrated microscope table). After the rotation of the mounting chamber, imaging was performed at the same distance from the proximal pipette.

We further verified our helix angle findings by a third experiment, digitally *flipping* image stacks (after deconvolution) along the *x*-axis. Results were as expected: a negation of $\varTheta _\mathrm{h}$ and $\varTheta _\mathrm{t}$, and $\varTheta _\mathrm{i}$ and both $\kappa$ parameters remaining unaltered. Therefore, it is unlikely that our statistically significant average *helix* angle findings are artefactual.

Historically, SMC orientation has been studied in a variety of different species and arteries. Some authors described a spiralling orientation of medial smooth muscle (Benninghoff 1927; Pflieger and Goerttler 1970; Schultze-Jena 1939; Strong 1938), whereas others described smooth muscle orientation as circumferential (Pichler et al. 1953; Ushiwata and Ushiki 1990). Benninghoff described already in 1927, a spiralling smooth muscle orientation with a helix angle increase towards the periphery (Benninghoff 1927). In 1939, Schultze-Jena assessed the muscle spiral in a variety of arteries and found opposing-handed helices in left and right arm arteries (Schultze-Jena 1939). A subsequent work by Pichler et al. (1953) assessed the potential mechanical consequences of a helical orientation. Pichler concludes, based on measurements in helically cut strips of carotid artery, cut at helix angles of $+30^\circ$ and $-30^\circ$ that a single-helical structure is unlikely. Although these historical studies provide excellent qualitative anatomical insight, only limited quantitative conclusions can be drawn.

The results we obtained, indicating a significant right-handed helix, contradict the conclusion of most quantitative studies that SMC orientation is strictly circumferential. In human cerebral arteries, Walmsley (1983) found a statistically non-significant mean helix angle of $-0.2 \pm 2.4^\circ$, in contrast to our angles of $2.4 \pm 2.2^\circ$ (inner layer) and $5.0 \pm 5.0^\circ$ (outer layer; $\mathrm{mean} \pm \mathrm{SD}$; left carotid arteries at $40 \, \mathrm{mmHg}$). Peters et al. reported a “truly circumferential” smooth muscle orientation and an “inconsequential” left- or right-handedness of the helix (Peters et al. 1983) in human cerebral arteries, whereas we found a consistent, significant helix, however, in viable carotid arteries of mice.

Some studies take a slightly different approach and consider the arterial (aortic) smooth muscle orientation to be a mixture of two or more distributions. Holzapfel et al., in their modelling paper in 2002, analysed SMC orientation in human aortas (Holzapfel et al. 2002). They implemented a method similar to our previous 2D method (Spronck et al. 2014b), but used histologically fixed instead of fresh tissue. Interestingly, Holzapfel et al. assumed the SMC orientation distribution to be centred around the circumferential direction, with two equally weighted helical parts. Using this assumption, they argued for two helices with angles of $\pm 8.4^\circ$. Tonar et al. (2015) assessed potential segmental differences in SMC orientation in porcine aortas, using a mixture of one to five Von Mises distributions. Their paper concludes that “The orientation of vascular smooth muscle was successfully fitted using two von Mises distributions in most of the samples...” and that “Only a minor fraction of samples required a tertiary von Mises component to describe the orientation...”. We carefully studied Supplemental Material 1 of their paper. When assessing the $N_\mathrm{c}=2$ columns (i.e. the columns that correspond to two Von Mises distributions), one can count that in 55 out of 82 samples, one of the two Von Mises components was weak and/or redundant. Tonar et al. (2015) state this in section 4.2 as “From a mathematical modelling point of view, this indicates that a structural model with a single vascular SMC system would be sufficient for most of our data”. Another finding of Tonar et al. is a segmental difference in SMC orientation dispersion (not in main orientation) along the aortic tree. This may also be the case along the carotid artery, potentially explaining the spread in $\kappa _1$ and $\kappa _2$ parameters we measured (whiskers in Figs. 7 and 8), since we may not have imaged the exact same part of the common carotid artery in all mice.

In our study, we analysed both left and right arteries and found a right-handed helix at both sites, violating left-right symmetry. Although very speculative, this may be explained by the intrinsic asymmetry of molecules, especially the proteins actin and myosin (Delhaas et al. 2008). As both molecules are arranged in a right-handed helix, when stretched, they will exhibit torsional motion. If a larger structure is built from such smaller helical components, an overall helicity may result. A more detailed discussion of this hypothesis can be found in Delhaas et al. (2008). In our case, when this hypothesis would hold, one would *expect* left and right arteries to show the same helical handedness, as we observed. It may also be the case that this molecular asymmetry effect acts through the extracellular matrix. As smooth muscle cells have been shown to align with the extracellular matrix collagen (O’Connell et al. 2008), asymmetry of the extracellular matrix will presumably translate to asymmetry in SMC orientations as well.

**Fig. 10:**
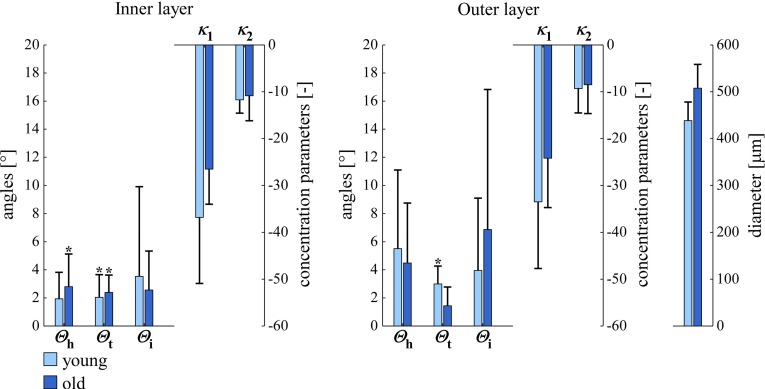
SMC orientations and arterial diameters in *left* carotid arteries at a luminal pressure of $40 \, \mathrm{mmHg}$ in young (age 8 weeks) and old (age 23 weeks) mice ($\mathrm{circular \, mean} \pm \mathrm{circular \, SD}$ for $\varTheta _\mathrm{h}$, $\varTheta _\mathrm{t}$, and $\varTheta _\mathrm{i}$; $\mathrm{arithmetic \, mean} \pm \mathrm{SD}$ for $\kappa _1$, $\kappa _2$, and diameter). $^{*}p<0.05$ versus angle zero (Rayleigh test). $\varTheta _\mathrm{h}$, $\varTheta _\mathrm{t}$, $\varTheta _\mathrm{i}$, $\kappa _1$, and $\kappa _2$ are defined in Fig. 6. *SD* standard deviation, *SMC* smooth muscle cell

Our study population of ten male C57BL/6JRj mice consisted of five young (age 8 weeks) and five older (age 23 weeks) individuals. We assessed whether age had an influence on orientations by comparing the results for the two age groups for left arteries at $40 \, \mathrm{mmHg}$ (Fig. 10) and found no statistically significant orientation parameter difference. We therefore decided to pool data from both age groups, yielding one group of $n=10$.

**Fig. 11:**
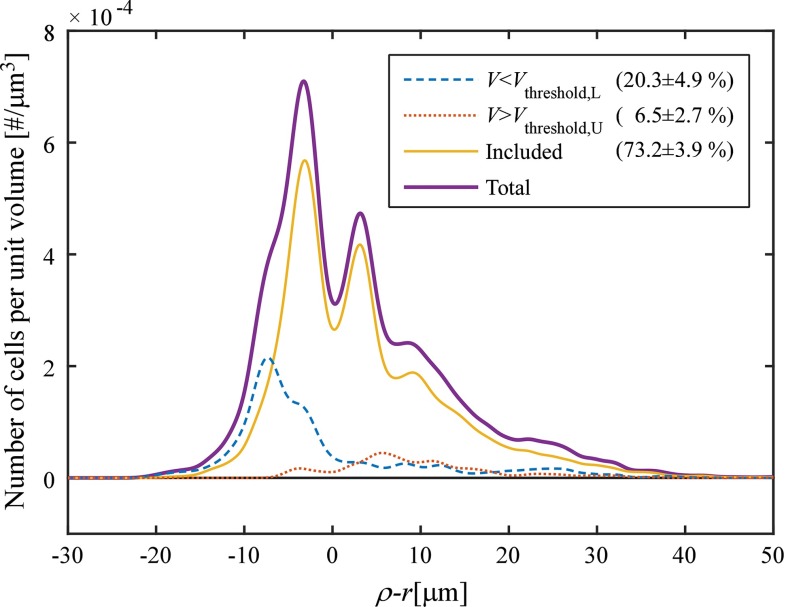
Effect of cluster volume thresholding. *Curves* show SMC density as a function of the relative radial coordinate ($\rho - r$), where $\rho$ is the radial coordinate as defined in Fig. 3a and *r* is half of the detected vessel diameter ($\frac{1}{2}d$). Pooled data of five samples at a luminal pressure of $40 \, \mathrm{mmHg}$ are shown [those (non-rotated) samples that were also used for assessing reproducibility (Table 1) and the effects of rotation (Fig. 9)]. The lower volume threshold ($V_\mathrm{threshold,L}$, *blue, dashed line*) removed clusters mainly at the inner side of the wall, whereas the upper volume threshold ($V_\mathrm{threshold,U}$, *red, dotted line*) removed clusters mainly at the outer side of the wall. Percentages pertain to the number of clusters in each stack that were excluded/included, respectively ($\mathrm{mean} \pm \mathrm{SD}$). *SD* standard deviation, *SMC* smooth muscle cell

Our study involved the use of lower and upper volume thresholds to exclude clusters that were too large or too small. The effect of this thresholding was analysed in a subset ($n=5$) of samples at $40 \, \mathrm{mmHg}$ (Fig. 11). Thresholding excluded $26.8 \pm 3.9\,\%$ (mean $\pm$ SD) of the clusters. The locations of these clusters are shown in Fig. 11. The combination of vesselness filtering and subsequent cluster volume thresholding was aimed at excluding non-SMCs. It should be noted that the number of nuclei excluded by vesselness filtering is not visible in Fig. 11. Calculating this number would require running the image analysis without vesselness filtering. However, vesselness filtering additionally functions as a means to convert the images from 12-bit greyscale ([0, 4095]) values to continuous ([0, 1]) values. Running the analysis without vesselness filtering, therefore, would require the definition of a new image threshold in the 12-bit domain, making comparison of the vesselness-filtered and vesselness-unfiltered results problematic.

Our finding of an arterial helix could have implications for the constitutive modelling of the artery wall. SMCs oriented in a slight net helix will create a slight torsion in the artery wall, which changes with SMC contraction. Such effects can and should be investigated using a constitutive modelling approach. Additionally, the fact that SMC orientation shows dispersion may have mechanical implications. An excellent example of the mechanical consequence of the inclusion of structural dispersion in constitutive models is given by the collagen modelling performed by Gasser et al. (2006). They found that, due to the dispersion of collagen, the recruitment of collagen fibres occurs at much lower stretches. In addition, when they excluded dispersion, collagen fibres showed larger than physiological rotations before bearing any load. Clearly, such effects also play a role in the modelling of SMC contraction in the artery wall.

Our study was limited in the sense that we assumed the orientation of the SMC nucleus to be representative of the orientation of the entire SMC. This assumption was also made in Holzapfel et al. (2002), Peters et al. (1983), Walmsley (1983). Staining nuclei instead of entire cells has the advantage that it leads to more robust clustering results. Canham et al. (1982) studied the coalignment of the muscle cell and nucleus and found the upper limit on misalignment in three dimensions to be $2.4^\circ$. We used an aspecific nuclear dye, staining nuclei of all cells in the artery wall. Therefore, non-SMCs (e.g. fibroblasts) may have been included in our analyses. However, by applying vesselness filtering, the narrow layer separation, and nucleus volume thresholds, we assume to have filtered out the majority of non-SMCs.

## Conclusions

We conclude that (1) vascular SMC orientation can be quantified in 3D; (2) SMC orientation shows considerable dispersion, predominantly in the helical direction, which decreases transversally with increasing luminal pressure; and (3) 3D quantification of SMC orientation reveals a distinct right-handed helical component in both left and right carotid arteries. These quantitative distribution data are essential to improve constitutive modelling of the artery wall.

## Figures and Tables

**Fig. 12:**
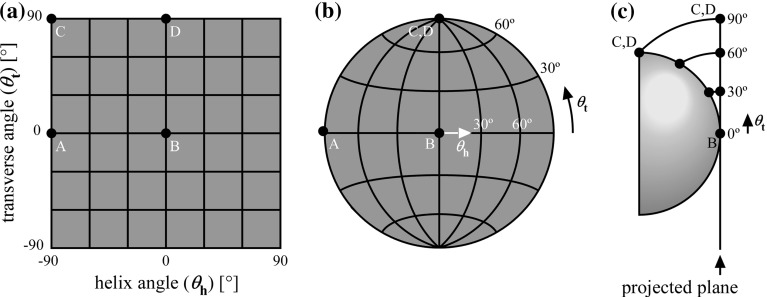
Considerations for using map projections. **a** Typical problem when representing a three-dimensional orientation without using a projection. Consider four points *A*, *B*, *C*, and *D* with indicated spherical coordinates. Points *A* and *B* clearly represent a different orientation that is $90^\circ$ apart. Points *C* and *D*, however, appear to also represent a different orientation, whereas in reality, they are equal (they are both at the “top” of the hemisphere). **b** The problem in **a** can be solved using a map projection. By representing the orientations using a projection, points *C* and *D* coincide. The projection used is the Lambert azimuthal equal-area projection. **c** A Lambert azimuthal equal-area projection is created by using a plane tangential to the centre point on the unit hemisphere (point B in this case). All points will be projected on this plane. From all points on the hemisphere, a circular arc is drawn to the projected plane that (1) ends perpendicular to the projected plane and (2) has point B as its centre

## Appendix 1: Projecting orientations on a unit hemisphere

The reader of this paper may wonder why a map projection is used to visualise SMC orientations. In this section, we will explain why the use of projections is beneficial.

An orientation in 3D can be quantified by two spherical angles: an elevation and an azimuth. In our study, the elevation corresponds to the transverse angle ($\theta _\mathrm{t}$) and the azimuth corresponds to the helix angle ($\theta _\mathrm{h}$). One could choose to make a rectangular plot and plot $\theta _\mathrm{h}$ versus $\theta _\mathrm{t}$. Gasser et al. (2012) have chosen to construct histograms in this way [Fig. 4 in Gasser et al. (2012)] and to depict an orientation density [Fig. 8 in Gasser et al. (2012)]. Although at first sight these representations appear feasible, they provide a deformed view of reality, for two (related) reasons:

1. Consider the surface element on the unit hemisphere spanning from $\theta _\mathrm{h}$ to $\theta _\mathrm{h} + \hbox { d}\theta _\mathrm{h}$ and $\theta _\mathrm{t}$ to $\theta _\mathrm{t} + \hbox { d}\theta _\mathrm{t}$. The area of this element is *not*$\hbox { d}\theta _\mathrm{h} \hbox { d}\theta _\mathrm{t}$, but $\hbox { d}\theta _\mathrm{h} \hbox { d}\theta _\mathrm{t} \cos \theta _\mathrm{t}$. Consequently, visualising orientations by using a rectangular $\theta _\mathrm{h}$ versus $\theta _\mathrm{t}$ plot over-emphasises orientations with large $\theta _\mathrm{t}$.
2. A related error occurs when trying to represent an orientation of $\theta _\mathrm{t} = \pm 90^\circ$. At what $\theta _\mathrm{h}$ should this orientation be drawn? Clearly, for $\theta _\mathrm{t} = \pm 90^\circ$, $\theta _\mathrm{h}$ is undefined.

These problems are illustrated in Fig. 12a. A solution to this problem could be to display the hemisphere in 3D, as performed by Alastrué et al. (2010). Although correct, such 3D figures are, to our opinion, hard to interpret from a (2D) document.

The problem of displaying spherical information on a flat surface is not new and occurs, for example, when creating a 2D map of the 3D world. The transformation from latitudes and longitudes of locations on the surface of a sphere into locations of a plane is called a map projection (Snyder and Voxland 1989). A large number of map projections exist, all suited for a specific goal. Importantly, there is not one general “best” projection (Snyder 1987). For our application, we require the projection to be equal area, tackling the aforementioned point 1 (Snyder 1987) and illustrated in Fig. 12b. Many projections possess this property, but the one that is most often used to plot directional data is the Lambert azimuthal equal-area projection (Borradaile 2003). This is the projection used in this paper. Its geometrical construction is explained in Fig. 12c. Mathematically, it is obtained via the following equations:

9 $$\begin{aligned} x&= \sqrt{\frac{2}{1+\cos \theta _\mathrm{t} \cos \theta _\mathrm{h}}} \cos \theta _\mathrm{t} \sin \theta _\mathrm{h}, \quad \hbox { and}\nonumber \\ y&= \sqrt{\frac{2}{1+\cos \theta _\mathrm{t} \cos \theta _\mathrm{h}}} \sin \theta _\mathrm{t} . \end{aligned}$$

## Appendix 2: Reproducibility

To evaluate our findings, in five arteries, we acquired a second image stack after physically rotated the mounting chamber by $180^\circ$ around the *z*-axis (see “Discussion” section and Fig. 9). As these rotated stacks were acquired at the same location as the corresponding non-rotated stacks, these data can be used to analyse the repeatability of our method.

**Table 1:** Reproducibility statistics

	$\mu$		$\sigma _\mu$		$\sigma _\mathrm{r}$	
	I	O	I	O	I	O
$\varTheta _\mathrm{h}$ ($^\circ$)	1.85	3.85	2.32	2.83	0.76	1.37
$\kappa _1$ (–)	$-25.80$	$-31.04$	7.75	8.44	2.37	6.33
$\kappa _2$ (–)	$-9.83$	$-9.66$	3.42	4.68	2.56	1.90
*d* (${\upmu \hbox {m}}$)	487		62		22	

$\mu$, overall mean; $\sigma _\mu$, between-animal standard deviation; $\sigma _\mathrm{r}$, reproducibility standard deviation. See “Appendix 2” for details on computing these values. $\varTheta _\mathrm{h}$, $\kappa _1$, and $\kappa _2$ are defined in Fig. 6. *d*, diameter; I and O, inner and outer layer, respectively

In order to assess repeatability, we calculated three summary values of parameters $P_{ij} \in \{\varTheta _{\mathrm{h},ij}, \kappa _{1,ij}, \kappa _{2,ij}, d_{ij}\}$, with $d_{ij}$ representing diameter. Subscript $i \in \{\mathrm{N},\mathrm{R}\}$ denotes non-rotated ($\mathrm{N}$)/rotated ($\mathrm{R}$) stacks; subscript $j \in \{1,2,3,4,5\}$ denotes animal number.

The following summary values were computed:

1. The overall mean ($\mu$):
  10 $$\begin{aligned} \mu = M\left[ m_1, m_2, ~\ldots , m_5 \right] ~. \end{aligned}$$
2. The between-animal standard deviation ($\sigma _\mu$):
  11 $$\begin{aligned} \sigma _\mu = S\left[ m_1, m_2, ~\ldots , m_5 \right] ~\mathrm{.} \end{aligned}$$
3. The reproducibility standard deviation ($\sigma _\mathrm{r}$):
  12 $$\begin{aligned} \sigma _\mathrm{r} =&\,S\left[ P_{\mathrm{N}1} \!-\! m_1, P_{\mathrm{R}1} \!-\! m_1, \right. \nonumber \\&P_{\mathrm{N}2} \!-\! m_2, P_{\mathrm{R}2} \!-\! m_2, \nonumber \\&\left. \ldots , P_{\mathrm{N}5} \!-\! m_5, P_{\mathrm{R}5} \!-\! m_5\right] . \end{aligned}$$

In these equations, *M* and *S* denote the circular mean and circular standard deviation operators for $P_{ij} = \varTheta _\mathrm{h,ij}$; and the arithmetic mean and standard deviation operators for $P_{ij} \in \{\kappa _{1,ij}, \kappa _{2,ij}, d_{ij}\}$, respectively. $m_j$ is used as a short-hand notation of $M (P_{\mathrm{N}j}, P_{\mathrm{R}j})$.

Results are shown in Table 1. In all cases, the reproducibility standard deviations were smaller than the between-animal standard deviations. Reproducibility of the helix angle ($\sigma _\mathrm{r}$) was good at $0.76^\circ$ (inner layer) and $1.37^\circ$ (outer layer), respectively.

## References

[CR1] Alastrué V, Sáez P, Martínez M, Doblaré M (2010). On the use of the Bingham statistical distribution in microsphere-based constitutive models for arterial tissue. Mech Res Commun.

[CR2] Benninghoff A (1927). Über die beziehungen zwischen elastischem gerüst und glatter muskulatur in der arterienwand und ihre funktionelle bedeutung. Z Zellforch Microsk Anat.

[CR3] Bingham C (1964) Distributions on the sphere and on the projective plane. Ph.D. thesis, Yale University

[CR4] Bingham C (1974). An antipodally symmetric distribution on the sphere. Ann Stat.

[CR5] Borradaile GJ (2003). Statistics of earth science data: their distribution in time, space and orientation.

[CR6] Canham PB, Henderson RM, Peters MW (1982). Coalignment of the muscle cell and nucleus, cell geometry and Vv in the tunica media of monkey cerebral arteries, by electron microscopy. J Microsc.

[CR7] Delhaas T, Kroon W, Bovendeerd P, Arts T (2008). Left ventricular apical torsion and architecture are not inverted in situs inversus totalis. Prog Biophys Mol Biol.

[CR8] Fisher N, Lewis T, Embleton B (1993). Statistical analysis of spherical data.

[CR9] Frangi A, Niessen W, Vincken K, Viergever M, Wells W, Colchester A, Delp S (1998). Multiscale vessel enhancement filtering. Medical image computing and computer-assisted interventation—MICCAI’98, vol 1496 of lecture notes in computer science.

[CR10] Gasser TC, Gallinetti S, Xing X, Forsell C, Swedenborg J, Roy J (2012) Spatial orientation of collagen fibers in the abdominal aortic aneurysm’s wall and its relation to wall mechanics. Acta Biomater 8(8):3091–3103. doi:10.1016/j.actbio.2012.04.044 10.1016/j.actbio.2012.04.044 22579983

[CR11] Gasser TC, Ogden RW, Holzapfel GA (2006). Hyperelastic modelling of arterial layers with distributed collagen fibre orientations. J R Soc Interface.

[CR12] Gonzalez RC, Woods RE (2008). Digital image processing.

[CR13] Holzapfel G, Gasser T, Stadler M (2002). A structural model for the viscoelastic behavior of arterial walls: continuum formulation and finite element analysis. Eur J Mech A Solid.

[CR14] Jähne B (1993). Spatio-temporal image processing: theory and scientific applications.

[CR15] Karshovska E, Zhao Z, Blanchet X, Schmitt MM, Bidzhekov K, Soehnlein O, von Hundelshausen P, Mattheij NJ, Cosemans JM, Megens RT, Koeppel TA, Schober A, Hackeng TM, Weber C, Koenen RR (2014) Hyperreactivity of junctional adhesion molecule a-deficient platelets accelerates atherosclerosis in hyperlipidemic mice. Circ Res. doi:10.1161/CIRCRESAHA.116.304035 10.1161/CIRCRESAHA.116.304035 25472975

[CR16] Mardia KV, Jupp PE (2000). Directional statistics.

[CR17] Masson I, Beaussier H, Boutouyrie P, Laurent S, Humphrey JD, Zidi M (2011). Carotid artery mechanical properties and stresses quantified using in vivo data from normotensive and hypertensive humans. Biomech Model Mechanobiol.

[CR18] Megens R, Reitsma S, Schiffers P, Hilgers R, De Mey J, Slaaf D, oude Egbrink M, van Zandvoort M (2007). Two-photon microscopy of vital murine elastic and muscular arteries. J Vasc Res.

[CR19] O’Connell MK, Murthy S, Phan S, Xu C, Buchanan J, Spilker R, Dalman RL, Zarins CK, Denk W, Taylor CA (2008). The three-dimensional micro- and nanostructure of the aortic medial lamellar unit measured using 3D confocal and electron microscopy imaging. Matrix Biol.

[CR20] Oheim M, Beaurepaire E, Chaigneau E, Mertz J, Charpak S (2001). Two-photon microscopy in brain tissue: parameters influencing the imaging depth. J Neurosci Methods.

[CR21] Onstott TC (1980). Application of the Bingham distribution function in paleomagnetic studies. J Geophys Res.

[CR22] Peters MW, Canham PB, Finlay HM (1983). Circumferential alignment of muscle cells in the tunica media of the human brain artery. Blood Vessels.

[CR23] Pflieger H, Goerttler K (1970). Konstruktionsprinzipien der aortenwand im ursprungsbereich der interkostalen, intestinalen und renalen aortenäste. Arch Kreislaufforsch.

[CR24] Pichler E, Lazarini W, Filippi R (1953). Über schraubenförmige struktur von arterien. Naunyn-Schmiedebergs Arch Pharmacol.

[CR25] Reitsma S, oude Egbrink MGA, Vink H, van den Berg BM, Passos VL, Engels W, Slaaf DW, van Zandvoort MAMJ (2011). Endothelial glycocalyx structure in the intact carotid artery: a two-photon laser scanning microscopy study. J Vasc Res.

[CR26] Schmitt MM, Megens RT, Zernecke A, Bidzhekov K, van den Akker NM, Rademakers T, van Zandvoort MA, Hackeng TM, Koenen RR, Weber C (2014). Endothelial junctional adhesion molecule-a guides monocytes into flow-dependent predilection sites of atherosclerosis. Circulation.

[CR27] Schober A, Nazari-Jahantigh M, Wei Y, Bidzhekov K, Gremse F, Grommes J, Megens RT, Heyll K, Noels H, Hristov M, Wang S, Kiessling F, Olson EN, Weber C (2014). Microrna-126-5p promotes endothelial proliferation and limits atherosclerosis by suppressing dlk1. Nat Med.

[CR28] Schultze-Jena B (1939). Über die schraubenförmige struktur der arterienwand. Gegenbauers Morphol Jahrbuch.

[CR29] Silverman BW (1986). Density estimation for statistics and data analysis.

[CR30] Slaaf DW, van Zandvoort MA (2011). Endothelial glycocalyx thickness and platelet-vessel wall interactions during atherogenesis. Thromb Haemostasis.

[CR31] Snyder JP (1987). Map projections—a working manual.

[CR32] Snyder JP, Voxland PM (1989). An album of map projections.

[CR33] Spronck B, Heusinkveld MH, Donders WP, de Lepper AG, Op’t Roodt J, Kroon AA, Delhaas T, Reesink KD (2015) A constitutive modeling interpretation of the relationship between carotid artery stiffness, blood pressure and age in hypertensive subjects. Am J Physiol Heart Circ Physiol 308:H568–H582. doi:10.1152/ajpheart.00290.2014 10.1152/ajpheart.00290.2014 25539709

[CR34] Spronck B, Megens R, Reesink K, Delhaas T (2014a) Three-dimensional vascular smooth muscle orientation as quantitatively assessed by multiphoton microscopy: mouse carotid arteries do show a helix. In: Conference Proceedings of the IEEE Eng Med Biol Soc IEEE, pp 202–205. doi:10.1109/EMBC.2014.6943564 10.1109/EMBC.2014.6943564 25569932

[CR35] Spronck B, Merken JJ, Reesink KD, Kroon W, Delhaas T (2014). Ureter smooth muscle cell orientation in rat is predominantly longitudinal. PLOS ONE.

[CR36] Strong KC (1938) A study of the structure of the media of the distributing arteries by the method of microdissection. Anat Rec 72(2):151–167. doi:10.1002/ar.1090720204

[CR37] Subramanian P, Karshovska E, Reinhard P, Megens RT, Zhou Z, Akhtar S, Schumann U, Li X, van Zandvoort M, Ludin C, Weber C, Schober A (2010) Lysophosphatidic acid receptors lpa1 and lpa3 promote cxcl12-mediated smooth muscle progenitor cell recruitment in neointima formation. Circ Res 107(1):96–105. doi:10.1161/CIRCRESAHA.109.212647 10.1161/CIRCRESAHA.109.212647 20360252

[CR38] Tanaka H (1999) Circular asymmetry of the paleomagnetic directions observed at low latitude volcanic sites. Earth Planets Space 51:1279–1286. doi:10.1186/BF03351601

[CR39] Tonar Z, Kochova P, Cimrman R, Perktold J, Witter K (2015). Segmental differences in the orientation of smooth muscle cells in the tunica media of porcine aortae. Biomech Model Mechanobiol.

[CR40] Ushiwata I, Ushiki T (1990). Cytoarchitecture of the smooth muscles and pericytes of rat cerebral blood vessels. J Neurosurg.

[CR41] Vader D, Kabla A, Weitz D, Mahadevan L (2009) Strain-induced alignment in collagen gels. PLOS ONE 4(6):e5902. doi:10.1371/journal.pone.0005902 10.1371/journal.pone.0005902 PMC2691583 19529768

[CR42] Walmsley JG (1983) Vascular smooth muscle orientation in straight portions of human cerebral arteries. J Microsc 131(Pt 3):361–375. doi:10.1111/j.1365-2818.1983.tb04261.x 10.1111/j.1365-2818.1983.tb04261.x 6355481

[CR43] Zulliger MA, Rachev A, Stergiopulos N (2004). A constitutive formulation of arterial mechanics including vascular smooth muscle tone. Am J Physiol Heart Circ Physiol.

